# Cerebellar transcranial direct current stimulation improves quality of life in individuals with chronic poststroke aphasia

**DOI:** 10.1038/s41598-025-90927-y

**Published:** 2025-02-26

**Authors:** Zhong Sheng Zheng, Jing Wang, Sharon Lee, Kevin Xing-Long Wang, Ben Zhang, Melissa Howard, Emily Rosario, Caroline Schnakers

**Affiliations:** 1 Research Institute, Casa Colina Hospital and Centers for Healthcare, 255 E. Bonita Ave, Pomona, CA 91767 USA; 2 Psychology Department, University of California, Los Angeles, CA USA

**Keywords:** Aphasia, Stroke, Transcranial direct current stimulation (tDCS), Cerebellum, Stroke and aphasia quality of life scale-39 (SAQOL-39), Psychosocial, Psychology, Medical research, Neurology

## Abstract

The cerebellum has emerged as a potential target for transcranial direct current stimulation (tDCS) in post-stroke aphasia (PSA) due to its role in language processing and relative preservation compared to supratentorial lesions. Recent evidence also highlights the cerebellum’s involvement in affective and social processes, suggesting potential broader effects of cerebellar modulation. This study investigated the efficacy of anodal tDCS over the right cerebellum paired with speech and language therapy in enhancing language functions and quality of life in individuals with PSA. Twenty-two participants with chronic PSA received cerebellar tDCS, while historical sham control data from 25 participants were obtained. Language outcomes were assessed using the Western Aphasia Battery-Revised (WAB-R), and secondary outcomes included patient-reported measures of communication effectiveness and quality of life. Mixed-design analyses of variance were conducted to examine treatment effects. No significant Group x Time interaction was found for WAB-R scores, indicating that tDCS did not provide additional language benefits over speech therapy. However, a significant Group x Time interaction was observed for the Stroke and Aphasia Quality of Life Scale-39 scores, driven by improvements in the Psychosocial, Physical, and Energy subdomains in the tDCS group. Cerebellar tDCS did not significantly improve language outcomes in PSA individuals but enhanced specific aspects of quality of life. These findings highlight the cerebellum’s multifaceted role in cognitive, affective, and sensorimotor processes. Future research should focus on conducting well-powered, randomized, double-blind, and concurrent trials to validate these findings and explore optimal stimulation parameters in PSA rehabilitation.

**Trial registration**: The trial is registered at ClinicalTrials.gov with the registration number NCT03699930. The date of registration is 10/05/2018.

**Supplementary Information:**

The online version contains supplementary material available at 10.1038/s41598-025-90927-y.

## Introduction

Aphasia, a language disorder characterized by deficits in the expression and/or comprehension of spoken and written language, is a common consequence of stroke^1^. Despite the availability of traditional speech and language therapy (SLT), many individuals with post-stroke aphasia (PSA) struggle to achieve satisfactory language outcomes. Non-invasive brain stimulation techniques, including transcranial magnetic stimulation (TMS) and transcranial direct current stimulation (tDCS), have emerged as promising adjunctive treatments for enhancing language recovery in PSA. TMS delivers electromagnetic pulses that can temporarily activate or inhibit specific brain regions^2^. An umbrella review by Georgiou et al. (2019) synthesized findings from systematic reviews on repetitive TMS (rTMS) and concluded that while rTMS shows potential in modulating cortical excitability for language recovery, the overall evidence remains inconclusive due to methodological limitations and variability across studies^3^. Additionally, rTMS requires specialized equipment and expertise, which may limit its accessibility in routine clinical settings.

In contrast, tDCS offers a simpler, portable, and more cost-effective alternative, making it a more feasible option for broader clinical implementation. TDCS applies weak electrical currents to modulate cortical excitability, where anodal stimulation increases excitability and cathodal stimulation decreases it^4^, thereby facilitating neuroplasticity and recovery. A systematic review by Biou et al. (2019) highlighted consistent improvements in naming, fluency, and overall communication outcomes in PSA patients receiving tDCS^5^. Similarly, a network meta-analysis by Elsner et al. (2020) demonstrated significant improvements in naming performance with anodal tDCS, particularly when applied to the left inferior frontal gyrus^6^. However, variability in study designs, stimulation protocols, and patient characteristics complicates efforts to establish standardized recommendations.

The majority of tDCS studies in PSA have focused on stimulating intact left perilesional regions, such as Broca’s area^7–9^, Wernicke’s area^10,11^, the motor cortex^12,13^, or the dorsolateral prefrontal cortex (dlPFC)^14^. While stimulation of Broca’s and Wernicke’s areas primarily aims to enhance linguistic processing, motor cortex stimulation has been associated with improvements in motor-speech functions and articulation, likely through its role in motor planning and execution for speech^12,13^. Moreover, recent findings suggest that dlPFC stimulation enhances higher-order cognitive processes, such as working memory, attention, and cognitive flexibility, which are critical for effective communication^15^. These studies suggest the interconnected nature of language, cognitive, and motor networks in post-stroke aphasia recovery.

Despite these advances, the efficacy of cortical stimulation may be limited by several factors. The presence of stroke lesions can lead to variable responses to direct stimulation over the damaged area, depending on the extent of the damage^16^. Moreover, the heterogeneity of supratentorial stroke lesions can make it challenging to determine the optimal stimulation site. While some studies have used functional MRI to guide tDCS targeting^17,18^, such approach is costly and not readily feasible in a clinical environment.

To address these challenges, the right cerebellum has been proposed as an alternative target for tDCS in post-stroke aphasia, as it is relatively intact compared to the supratentorial regions primarily affected in PSA. The right cerebellum has been associated with a host of cognitive and language functions^19–21^, and both functional and structural connectivity studies have identified connections between the right cerebellum and contralateral fronto-temporo-parietal language association areas^22–25^.

Promising results have been observed in both healthy volunteers and individuals with PSA following cerebellar tDCS. In healthy participants, cerebellar tDCS has led to improvements in verbal fluency^16^ and verb generation^26^. Similarly, in post-stroke aphasia, cerebellar tDCS has been shown to enhance spelling^27^, naming^28,29^, and verb generation^30^. Furthermore, increased right cerebellar activation has been linked to better recovery in patients with aphasia^31^, suggesting that the right cerebellum may play a crucial role in language recovery post-stroke. The mechanisms underlying the effects of cerebellar tDCS on language processing are thought to involve the modulation of Purkinje cells^26,32^, which are the primary output neurons of the cerebellar cortex and regulate the activity of deep cerebellar nuclei. By altering the excitability of Purkinje cells, cerebellar tDCS may induce changes in the firing patterns of the deep cerebellar nuclei, which in turn can influence the activity of distant cortical areas through cerebello-cortical projections^32,33^. This is supported by our prior work, which uncovered neuroplasticity in the superior cerebellar peduncle, a major white matter tract carrying efferent fibers from the deep cerebellar nuclei, associated with improvement in spontaneous speech following anodal Broca’s tDCS in patients with PSA^34^. These findings suggest that modulating cerebellar activity, either directly through cerebellar tDCS or indirectly through cortical stimulation, may promote language recovery by inducing neuroplastic changes in the cerebello-cortical network.

Beyond its established role in motor control, emerging evidence highlights the cerebellum’s involvement in language processing as well as affective and social cognition^23,35^. Neuroimaging studies have revealed cerebellar activation during tasks involving emotion processing, empathy, mentalizing or theory of mind, and social interaction^36–40^. Lesion studies have shown that cerebellar damage can lead to cerebellar cognitive affective syndrome, characterized by impairments in affective regulation, executive function, linguistic processing, and visuospatial cognition^41,42^. Moreover, the cerebellum has been found to play a role in the regulation of mood and anxiety, with structural and functional cerebellar abnormalities observed in various psychiatric disorders^43,44^. The cerebellum’s dense connections with limbic and paralimbic regions, including the amygdala, hypothalamus, hippocampus, cingulate cortex, and medial prefrontal cortex, provide an anatomical basis for its involvement in emotional, social, and higher-order cognitive processing^23,45^. These diverse cerebellar functions suggest a potential for wide-ranging effects of cerebellar modulation beyond motor and language domains. However, the impact of cerebellar tDCS on this broad spectrum of functions in individuals with post-stroke aphasia remains largely unexplored.

Targeting the more preserved cerebellum may offer a more consistent and practical approach to addressing heterogeneous stroke lesions. While previous studies have investigated the effects of cerebellar tDCS on language production, its potential to enhance a broader range of language abilities (e.g., language comprehension) and its associated functions remains to be elucidated. The primary objective of this study was to evaluate the efficacy of anodal tDCS over the right cerebellum in enhancing both expressive and receptive language functions, as assessed by the Western Aphasia Battery-Revised (WAB-R) in individuals with chronic PSA. Additionally, given the cerebellum’s involvement in various cognitive and affective processes, we aimed to explore the effects of cerebellar tDCS on patient-reported secondary outcomes, particularly quality of life and communication effectiveness. To provide a robust evaluation of the intervention effects, the results were compared to a previous sham control dataset.

## Methods and materials

### Participants

This study included 47 participants with chronic post-stroke aphasia, primarily resulting from left hemisphere strokes, with some participants exhibiting bilateral lesions. Lesion information for each participant is shown in Supplementary Table S1. MRI scans were not available from some patients; thus, lesion details were only available for 21/22 tDCS patients and 19/25 sham patients. The participants were divided into two groups: the cerebellar tDCS condition (*n* = 22) and sham condition (*n* = 25). We initially recruited 25 patients for cerebellar tDCS, but three dropped out due to either COVID-19 infection, schedule conflict, or lack of English proficiency. The sample size for the cerebellar tDCS group was chosen to align with the sham dataset from a previous study (Zheng et al., 2024), ensuring consistency and direct statistical comparisons between the groups, while adhering to the parameters established in the prior study to detect meaningful differences in language outcomes. The cerebellar tDCS dataset were acquired between 2020 and 2022 with a non-randomized, double-blind design. The sham tDCS data were obtained between 2018 and 2020, which used a randomized, double-blind design. Table 1 summarizes the participants’ demographic and clinical information (see Supplementary Table S2 for each participant’s detailed information). The inclusion criteria for participants were as follows: (1) a clinical diagnosis of aphasia resulting from ischemic or hemorrhagic stroke, (2) at least 12 months post-stroke, (3) aged 18 years or older, and (4) English proficiency (native and non-native speakers). English proficiency was evaluated based on participants’ functional ability to engage in the study protocol. Specifically, during the in-person assessment, the speech-language pathologist determined whether the participants could understand and follow testing instructions and demonstrate basic conversational skills in English, taking into consideration their aphasia severity. Participants with multiple strokes were also included. Exclusion criteria consisted of aphasia resulting from any neurological conditions other than stroke. All participants provided written informed consent prior to participating in the study. The study was approved by the Institutional Review Board of Casa Colina Hospital. The trial was registered on ClinicalTrials.gov as NCT03699930.

**Table 1:** Patient demographic and clinical details.

Variable	cTDCS	Sham
N	22	25
Age (years)	63.4 ± 12.7	64 ± 11.1
Time since Injury (months)*	30.6 ± 20.3	87.7 ± 74.8
Aphasia quotient (baseline)	50.6 ± 27.1	60.3 ± 28.5
Male/female	17/5	20/5
Ischemic/hemorrhagic/both	17/3/2	20/3/2
Fluent/non-fluent	9/13	13/12
Left/right handed	3/19	1/24
Left only/bilateral lesion/NA	15/6/1	13/6/6

The table summarizes participant demographics and clinical characteristics, including age, time since injury, baseline aphasia quotient, gender distribution, stroke type (ischemic, hemorrhagic, or both), fluency (fluent or non-fluent aphasia), handedness (left- or right-handed), and lesion location (left hemisphere, bilateral, or not applicable [MRI not available]). Data are presented as mean ± standard deviation for continuous variables and counts for categorical variables. The cTDCS group included 22 participants, while the sham group included 25 participants.

*Time since injury was significantly shorter in the cTDCS group compared to the Sham group (*p* < 0.05), and this variable was included as a covariate in ANOVA analyses.

### Study design

Following a double-blind design, both participants and experimenters who conducted the interventions and assessments were blinded to the allocation of participants. Participants in the cerebellar tDCS group were allocated using a non-randomized, double-blind design. Allocation concealment was achieved by assigning each participant a unique six-digit code, with only the principal investigator (PI) aware of group allocation to maintain blinding of both participants and experimenters. For the sham group, data were drawn from a previous study that employed a randomized, double-blind design to assign participants to sham stimulation^46^. To incorporate the pre-existing control data while preserving the study’s blinded nature, participants in the active tDCS group were told they could receive either real cerebellar tDCS or sham tDCS paired with speech and language therapy; however, all participants in the active group received real tDCS.

For both the sham and cerebellar tDCS groups, the primary outcome measure was collected at two time-points, within one week before the intervention (pre-intervention) and one week after the intervention (post-intervention). Secondary outcome measures were collected at three time points: pre-intervention, post-intervention, and during a 3-month follow-up.

### Outcome measures

The primary outcome measure was change in language scores as assessed by the Western Aphasia Battery-Revised (WAB-R). This evaluation encompassed four distinct subscales, namely spontaneous speech (SS), auditory verbal comprehension (AVC), name and word finding (NWF), and repetition (REP). The scores from these four subscales were consolidated to compute the aphasia quotient (AQ [range 0-100]), providing a measure of aphasia severity, with lower scores reflecting worse language performance. Secondary outcome measures (SOMs) were patient and/or caregiver self-reported and comprised the Communication Outcomes after Stroke (COAST), Carer COAST, Stroke and Aphasia Quality of Life Scale-39 (SAQOL-39), and Patient-Reported Outcomes Measurement Information System (PROMIS-Global). Higher scores indicate better outcomes. The COAST and Carer-COAST surveys consist of 20 items evaluating post-stroke communication effectiveness. The PROMIS-Global (10 items) and SAQOL-39 (39 items) evaluate patients’ quality of life and overall well-being. The SAQOL-39 assesses functioning in four domains: physical, psychosocial, communication, and energy.

### Transcranial direct current stimulation

TDCS stimulation was paired with speech and language therapy and was delivered for 20 min using a constant current stimulator (Soterix Medical 1 × 1 clinical trials device). Consistent with other studies on cerebellar tDCS^29,47^, the current study utilized 2 mA tDCS generated between two 5 cm x 5 cm saline-soaked sponges. The anode (active electrode) was placed on the right cerebellar cortex, 1 cm under, and 4 cm lateral to the inion (approximately over the cerebellar lobule VII)^26^. The reference electrode (cathode) was placed on the right deltoid. The current was ramped up to 2 mA over 30 s, maintained for 20 min, then ramped down over 30 s.

In the sham condition (data previously collected), after the initial ramp-up period of 30 s, the intensity was promptly reduced to 0 mA in the subsequent 30-second ramp-down phase and maintained at this level throughout the session. Each stimulation condition, whether real or sham, was coupled with a 20-minute speech and language therapy session. Participants underwent this procedure for five consecutive days.

### Speech and language therapy

Participants underwent approximately 20 min of personalized speech and language therapy, tailored by the therapist’s clinical judgment, and guided by standardized assessments. The primary goal of the therapy was to enhance expressive language skills, employing a range of evidence-based treatment techniques, such as Melodic Intonation Therapy, Language Stimulation Approach, Modified Response Elaboration Training, Semantic Feature Analysis Treatment, Verb Network Strengthening Treatment, Sound Production Treatment, Integral Stimulation, and Word Retrieval Cuing Strategies (e.g., phonological and semantic cuing). For participants with severe auditory comprehension deficits that impeded their ability to understand tasks, the therapy incorporated targeted auditory comprehension interventions. The therapy sessions prioritized areas that would yield the most significant improvements in the participant’s functional communication skills.

### Statistical analysis

To identify potential confounding variables, we compared the demographic and clinical characteristics of the two groups, including age, gender, time since injury (TSI), stroke type (ischemic or hemorrhagic), fluency (fluent or non-fluent), baseline AQ, handedness, and lesion hemisphere. Independent samples t-tests were used for continuous variables, while chi-square tests were employed for categorical variables. TSI was found to be significantly different between the two groups and was therefore included as a covariate in subsequent analyses.

Mixed-design analyses of variance (ANOVAs) were conducted to examine the treatment effects, with Group (tDCS or sham) as the between-subjects factor and Time (pre-intervention or post-intervention) as the within-subjects factor. The primary outcome measure consisted of scores from WAB-R, including AQ, SS, AVC, NWF, and REP. Secondary outcomes included scores from COAST, Carer COAST, PROMIS-Global, and SAQOL-39.

After finding a significant Time X Group interaction for SAQOL-39 scores, we conducted a follow-up analysis (Mixed ANOVAs with TSI as a covariate) to determine which subdomains (Physical, Psychosocial, Communication, Energy) of SAQOL-39 were driving the significance. To control for multiple comparisons, a False Discovery Rate (FDR) correction was applied at a threshold of q = 0.05, balancing the control of Type I error with sufficient sensitivity to detect meaningful effects. Due to the limited number of questions (four) in the Energy subdomain, which may have resulted in a restricted range of scores and reduced variability, we combined the Energy and Physical subdomain scores. Three participants were missing SAQOL-39 data, resulting in *n* = 20 for tDCS and *n* = 24 for sham.

Due to challenges faced during the COVID-19 pandemic, many participants were unable to complete the 3-month follow-up assessment, resulting in insufficient data for group analysis at this time point. Consequently, only pre-intervention and post-intervention scores were included in the statistical analyses.

## Results

**Fig. 1:**
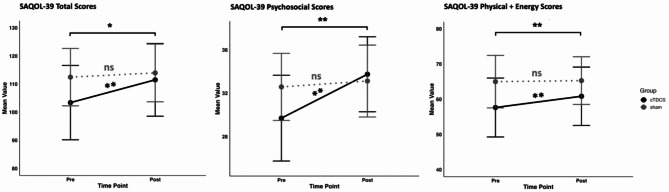
SAQOL-39 Interaction Graphs. The graphs depict significant interactions between group and time for the SAQOL-39 Total Scores, Psychosocial Scores, and Physical + Energy Scores. Mean values for each group (cTDCS = solid black; sham = dotted gray) are shown at both pre- and post-intervention time points. Error bars represent 95% confidence intervals. *ns* non-significant; * = p $\:\le\:$ 0.05; ** = p $\:\le\:$ 0.01; *** = p $\:\le\:$ 0.001.

The two groups did not differ significantly in age, gender, stroke type, fluency, or AQ at baseline, but did differ in time since injury (Table 1); the tDCS group had significantly shorter TSI compared to the sham group. Therefore, TSI served as a covariate in the ANOVA analyses. The ANOVA results revealed no Group x Time interaction for the WAB-R scores, indicating that the change in language performance over time did not differ between the tDCS and sham groups (Table 2). We, did, however, find a significant Group x Time interaction (*F*(1,41) = 5.36, *P* = 0.026, $\:{\eta\:}^{2}$ = 0.005) for SAQOL-39 (total scores) (Fig. 1). The tDCS group showed a significant increase in SAQOL-39 scores from pre- to post-intervention (mean change = 8.7, SD = 10), while the sham group did not (mean change = 1.5, SD = 12). Further analysis of the SAQOL-39 subdomains revealed significant Time X Group interactions for both Psychosocial (*F*(1,41) = 6.61, *P* = 0.014, $\:{\eta\:}^{2}$ = 0.012) and Physical + Energy subdomains (*F*(1,41) = 5.79, *P* = 0.021, $\:{\eta\:}^{2}$ = 0.003); see Fig. 1. The tDCS group demonstrated significant increases in Psychosocial (mean change = 4.1, SD = 5) and Physical + Energy (mean change = 3.2, SD = 4.4) from pre- to post-intervention, whereas the sham group showed no significant changes (Psychosocial: mean change = 0.54, SD = 4.3; Physical + Energy: mean change = 0.29, SD = 6.9). No significant differences were found between the groups for the Communication subdomain. No other secondary outcomes showed significant differences between groups after treatment. No severe adverse events were reported in either group.

**Table 2:** WAB-R scores from pre- to post-treatment.

	cTDCS			Sham		
WAB-*R*	Pre	Post	Post-Pre	Pre	Post	Post-Pre
AQ	50.6 ± 27	53.6 ± 26.4	3 ± 2.7	60.3 ± 28.5	63.6 ± 28.5	3.3 ± 4.3
SS	9 ± 5.7	9.8 ± 5.3	0.7 ± 1.3	11.2 ± 5.9	12.1 ± 5.8	0.96 ± 2
AVC	7.1 ± 2.1	7.3 ± 2.3	0.19 ± 0.5	7.6 ± 2.5	7.7 ± 2.5	0.7 ± 0.4
REP	5.09 ± 3.5	5.1 ± 3.4	0.02 ± 0.6	5.6 ± 3.4	5.8 ± 3.3	0.2 ± 0.4
NWF	4 ± 3.4	4.6 ± 3.5	0.58 ± 0.7	5.7 ± 3.3	6.1 ± 3.3	0.4 ± 0.5

Data are presented as mean ± standard deviation. The graphs depict significant interactions between group and time for the SAQOL-39 Total Scores, Psychosocial Scores, and Physical + Energy Scores. Mean values for each group (cTDCS = solid black; sham = dotted gray) are shown at both pre- and post-intervention time points. Error bars represent 95% confidence intervals.

*WAB-R* Western aphasia battery-revised;* AQ* aphasia quotient;* SS* spontaneous speech,* AVC* auditory verbal comprehension,* REP* repetition,* NWF* name and word finding, *ns* non-significant.

* = p $$\:\le\:$$ 0.05; ** = p $$\:\le\:$$ 0.01; *** = p $$\:\le\:$$ 0.001.

## Discussion

This study aimed to investigate the effectiveness of anodal tDCS over the right cerebellum in enhancing language functions in individuals with post-stroke aphasia. Despite the growing interest in the cerebellum as a potential target for neuromodulation in aphasia treatment, our results did not demonstrate significant improvements in language processing, as measured by the WAB-R, in the cerebellar tDCS group compared to the sham group. The lack of significant interaction effects between Group and Time on the primary outcome measure (WAB-R scores) suggests that cerebellar tDCS did not provide additional benefits over traditional speech and language therapy alone. This finding is inconsistent with our initial hypothesis and some previous studies that have reported positive effects of cerebellar tDCS on language functions^27,29,47^. However, it is important to note that the current literature on cerebellar tDCS in aphasia treatment is still limited, and the optimal stimulation parameters and treatment protocols remain unclear.

Despite the lack of significant effects on the WAB-R scores, we found a significant Group x Time interaction for the SAQOL-39 total scores, driven by improvements in Psychosocial and Physical + Energy subdomains. The cerebellar tDCS group showed a significant increase in SAQOL-39 scores from pre- to post-intervention, while the sham group did not. These findings suggest that cerebellar tDCS may have potential benefits for enhancing specific aspects of quality of life in individuals with PSA, even in the absence of measurable changes in language performance.

The improvement in the Psychosocial subdomain may be attributed to the role of the cerebellum in social cognition and emotional processing^38,48^. The posterior cerebellum, particularly the lateral region targeted by our tDCS intervention, has been implicated in complex cognitive-social processes such as mentalizing, emotion attribution, and social judgment^38,49,50^. A meta-analysis by Van Overwalle et al. (2014) found consistent activation in the posterolateral cerebellum during various social and cognitive tasks^37^. Moreover, anodal cerebellar tDCS applied to the posterior cerebellum has been shown to improve the ability to recognize and attribute mental states to others^51^. These findings are further supported by preclinical studies, where stimulating the right posterolateral cerebellum in an autism mouse model led to improvements in social behaviors^52^.

In addition to its role in social cognition, the cerebellum has reciprocal monosynaptic connections with the hypothalamic-pituitary-adrenal (HPA) axis^53,54^, a key component of the neuroendocrine system that regulates the body’s response to stress. Dysregulation of the HPA axis has been linked to various psychiatric disorders, which are often associated with impaired psychosocial functioning^55^. Neuroimaging studies have found structural and functional abnormalities in the cerebellum of patients with various psychiatric and stress-related disorders^44,56–58^. Given the cerebellum’s involvement in both social cognition and stress regulation, cerebellar tDCS may have modulated the neural networks involved in processing social cues and regulating emotional responses, leading to better psychosocial functioning and well-being in individuals with PSA.

Following the discussion on the cerebellum’s role in social cognition and emotional regulation, it is important to note that the posterior cerebellum is also critically involved in sensorimotor functioning^59^, albeit to a lesser extent than the anterior cerebellum. While the anterior cerebellum is primarily associated with basic motor control, the posterior cerebellum may contribute to more complex aspects of motor control and planning that integrate cognitive and affective processes^22,23^. Functional neuroimaging studies have shown activation of the posterior cerebellum during various sensorimotor tasks^60–64^. Moreover, stimulating the posterior cerebellum may also have indirectly influenced the anterior cerebellum through the extensive network of cerebellar connections^22^. Cerebellar tDCS may have enhanced the efficiency of neural networks responsible for sensorimotor control, resulting in increased physical functioning and energy levels, aligning with cerebellum’s well-established role in motor functioning.

Interestingly, the Communication subdomain did not show significant differences between the tDCS and sham groups, which is consistent with the lack of significant effects on the primary language outcome measures (WAB-R scores). This suggests that while cerebellar tDCS may have the potential to improve certain aspects of quality of life, its direct impact on language functions remains unclear. The differential effects of cerebellar tDCS on the SAQOL-39 subdomains highlight the need for future research to investigate the specific mechanisms through which cerebellar stimulation may influence different aspects of functioning and well-being in individuals with aphasia. Moreover, the development of targeted intervention protocols that combine cerebellar tDCS with focused rehabilitation strategies for psychosocial and physical functioning may help to optimize the potential benefits of this neuromodulation technique.

In a previous clinical trial, we investigated the efficacy of tDCS applied over Broca’s area compared to sham stimulation in individuals with post-stroke aphasia^34^. While the study collected SAQOL-39 scores, no significant differences were found between the Broca’s tDCS and sham groups in terms of quality of life outcomes. In contrast, the current study demonstrated significant improvements in SAQOL-39 scores, particularly in the Psychosocial and Physical + Energy subdomains, following cerebellar tDCS compared to sham stimulation. These findings suggest that targeting the cerebellum with tDCS may have unique potential in enhancing quality of life outcomes in PSA, possibly due to its role in modulating cognitive, affective, and sensorimotor processes that extend beyond language-specific functions. The lack of significant effects on SAQOL-39 scores when targeting Broca’s area highlights the specific contribution of the cerebellum to these quality of life improvements.

The significant improvement in SAQOL-39 scores for the cerebellar tDCS group, in the absence of significant changes in other secondary outcome measures, may be attributed to the comprehensive nature of the SAQOL-39. This 39-item scale assessed multiple domains, which may have enabled it to capture subtle changes in participants’ overall well-being and functioning. In contrast, the COAST (20 items) and PROMIS-Global (10 items) are shorter scales focusing on specific aspects of communication or quality of life. The broader scope and higher number of items in the SAQOL-39 may have increased its sensitivity to detect minor improvements in quality of life that were not captured by the other secondary outcome measures.

However, it is important to acknowledge the limitations of this study. One major limitation of the study is the difference in data collection periods between the two groups. The cerebellar tDCS data were collected during the COVID-19 pandemic, which could have introduced additional confounding factors. For instance, participants in the cerebellar tDCS group wore clear masks during treatment sessions to comply with pandemic safety protocols, while participants in the sham group, whose data were acquired before the pandemic, did not. The use of clear masks might have affected the quality of communication and the effectiveness of speech and language therapy, resulting in null results for the primary outcome. Additionally, psychosocial factors related to the pandemic, such as changes in participants’ mental health, social support, and access to healthcare services could have influenced the participants’ performance and response to treatment. Furthermore, the non-randomized design for the cerebellar tDCS group may have introduced selection bias and reduced the comparability between the two groups.

Another limitation of the study is the relatively short duration and non-standardized nature of the therapy sessions. The 20-minute sessions may not have provided sufficient time for patients to engage in intensive, consistent language therapy necessary to induce measurable changes in language performance, especially considering the chronic nature of the participants’ aphasia. Personalized therapy reflects real-world clinical practice but may have introduced variability in therapy delivery, reducing the consistency of the intervention across patients. Details of the specific types of speech and language therapy each patient received are listed in Supplementary Table S2.

Furthermore, the WAB-R, while a widely used and validated assessment tool for aphasia, might not have been sensitive enough to detect small improvements in specific language domains, particularly when combined with the variability in therapy approaches and the relatively short intervention period. Extending the intervention to a longer duration, while implementing more standardized and focused language therapy protocols and using more sensitive assessment tools, could have facilitated more pronounced improvements in language outcomes. However, logistical constraints and feasibility concerns influenced our decision not to extend the therapy duration. Longer treatment protocols could have posed challenges for participant retention, increasing the risk of dropout and reducing the statistical power of the study. Moreover, the sham data were collected in an earlier study using the same 5-day protocol, and maintaining consistency in study design ensured meaningful comparisons between the cerebellar tDCS and sham groups.

Finally, the inclusion of non-native English speakers could have potentially influenced the linguistic results, as language proficiency may impact both baseline performance and responsiveness to therapy. Bilingual or non-native speakers often display distinct patterns of language recovery due to cross-linguistic transfer and differences in neural organization of language networks^65,66^. Future studies should consider stratifying patients based on language background and age of English acquisition to better understand how these factors might influence response to intervention.

In conclusion, our study did not provide evidence for the efficacy of cerebellar tDCS in improving language functions in individuals with chronic post-stroke aphasia. However, the significant improvement in SAQOL-39 scores, particularly in the Psychosocial and Physical + Energy subdomains, suggests potential benefits of cerebellar tDCS group on participants’ quality of life. These findings highlight the cerebellum’s multifaceted role in modulating cognitive, affective, and sensorimotor processes. While these results are promising, they should be interpreted with caution due to the study’s limitations, including the non-randomized design and the potential confounding effects of COVID-19 pandemic. Future research should focus on conducting larger-scale, parallel, randomized controlled trials with longer follow-up periods to establish the clinical utility of cerebellar tDCS in aphasia treatment. Additionally, employing more sensitive and targeted outcome measures, as well as exploring optimal stimulation parameters and treatment protocols, may help to better understand the potential of cerebellar tDCS in enhancing language recovery and quality of life in individuals with post-stroke aphasia.

## Electronic supplementary material

Below is the link to the electronic supplementary material.

Supplementary Material 1

Supplementary Material 2

## Data Availability

Due to privacy and ethical considerations, data are only available upon request by contacting the corresponding author, Z.S.Z., at azheng@casacolina.org.
