# Supplementary material for: Cerebellar transcranial direct current stimulation improves quality of life in individuals with chronic poststroke aphasia
Source: Sci Rep. 2025 Feb 26;15:6898. doi: 10.1038/s41598-025-90927-y (PMC11865529; doi:10.1038/s41598-025-90927-y)
Supplement: Supplementary file 1 — Supplementary Material 1 [file 41598_2025_90927_MOESM1_ESM.docx]

**Supplementary Table S1. Lesion information for each participant**

| **ID** | **Group** | **Lesion Information** |
| --- | --- | --- |
| 1 | cTDCS | Left basal ganglia, thalamus, external capsule, cerebral peduncle |
| 2 | cTDCS | Left parietal, cerebral peduncle, pons. Right anterior corona radiata, external capsule |
| 3 | cTDCS | Left frontal, temporal, cerebral peduncle |
| 4 | cTDCS | Left frontal, temporal, parietal, occipital, basal ganglia |
| 5 | cTDCS | Bilateral corona radiata |
| 6 | cTDCS | Left frontal, parietal |
| 7 | cTDCS | Left posterior limb of internal capsule, cerebral peduncle |
| 8 | cTDCS | Left frontal, temporal |
| 9 | cTDCS | Left frontal, temporal, parietal |
| 10 | cTDCS | Left frontal, occipital. Right frontal |
| 11 | cTDCS | Left thalamus |
| 12 | cTDCS | Left temporal, parietal |
| 13 | cTDCS | Left frontal, temporal, parietal, cerebral peduncle |
| 14 | cTDCS | Bilateral corona radiata, basal ganglia. Left thalamus |
| 15 | cTDCS | Left frontal, temporal, parietal |
| 16 | cTDCS | Left frontal, temporal, parietal |
| 17 | cTDCS | Left frontal, temporal, parietal, occipital. Right parietal, occipital |
| 19 | cTDCS | N/A |
| 20 | cTDCS | Left frontal, temporal, parietal, occipital, cerebral peduncle |
| 21 | cTDCS | Bilateral corona radiata. Left thalamus, pontomedullary junction. Right cerebellum |
| 22 | cTDCS | Left temporal, parietal, occipital |
| 23 | cTDCS | Left frontal, temporal, parietal, basal ganglia |
| 22 | sham | Left frontal, parietal, basal ganglia, corona radiata, external capsule, cerebral peduncle, pons. Right cerebellum |
| 23 | sham | Left occipital. Right basal ganglia, cerebellum |
| 24 | sham | Left frontal, temporal, parietal, basal ganglia, midbrain |
| 25 | sham | Left frontal, temporal, parietal, occipital, basal ganglia |
| 26 | sham | N/A |
| 27 | sham | Left frontal, temporal. Right frontal |
| 28 | sham | Left frontal, temporal, parietal, basal ganglia, cerebral peduncle |
| 29 | sham | Left frontal, insula, basal ganglia |
| 30 | sham | Left frontal, temporal, parietal, occipital, basal ganglia, midbrain |
| 31 | sham | N/A |
| 32 | sham | N/A |
| 33 | sham | N/A |
| 34 | sham | Left frontal, temporal, parietal, occipital, thalamus, pons. Right frontal, temporal |
| 35 | sham | Left frontal, parietal, occipital |
| 36 | sham | N/A |
| 37 | sham | Left frontal, temporal, insula, basal ganglia, corona radiata. Right temporal |
| 38 | sham | Left frontal, temporal, basal ganglia |
| 39 | sham | Left frontal, temporal, parietal, basal ganglia, cerebral peduncle |
| 40 | sham | Left frontal, temporal, parietal, basal ganglia, cerebral peduncle |
| 41 | sham | Left frontal, parietal |
| 42 | sham | Left temporal, insula, basal ganglia |
| 43 | sham | Left frontal, pons |
| 44 | sham | Left frontal, temporal, parietal, occipital, basal ganglia, midbrain. Right cerebellum |
| 45 | sham | N/A |
| 46 | sham | Left frontal, temporal, parietal, occipital. |
