# Supplementary material for: Cerebellar transcranial direct current stimulation improves quality of life in individuals with chronic poststroke aphasia
Source: Sci Rep. 2025 Feb 26;15:6898. doi: 10.1038/s41598-025-90927-y (PMC11865529; doi:10.1038/s41598-025-90927-y)
Supplement: Supplementary file 2 — Supplementary Material 2 [file 41598_2025_90927_MOESM2_ESM.docx]

| **ID** | **Group** | **Age** | **TSI (Days)** | **AQ (Baseline)** | **Gender** | **Stroke Type** | **Fluency** | **Handedness** | **Therapy Methods** |
| --- | --- | --- | --- | --- | --- | --- | --- | --- | --- |
| 1 | cTDCS | 51 | 794 | 64.7 | Male | Ischemic | Nonfluent | Right | SFAT, VNST, WRC, ACT |
| 2 | cTDCS | 67 | 383 | 53.1 | Male | Ischemic | Nonfluent | Left | WRC |
| 3 | cTDCS | 53 | 762 | 68.2 | Female | Ischemic | Fluent | Right | SFAT, VNST, WRC |
| 4 | cTDCS | 72 | 465 | 12 | Male | Ischemic | Nonfluent | Left | MIT, ACT |
| 5 | cTDCS | 68 | 970 | 84.8 | Male | Hemorrhagic | Fluent | Right | LSA, MRET, SFAT, WRC |
| 6 | cTDCS | 51 | 383 | 60.9 | Female | Ischemic | Fluent | Right | MRET, VNST, WRC |
| 7 | cTDCS | 38 | 1301 | 58.7 | Male | Ischemic | Nonfluent | Right | SFAT, IS, WRC |
| 8 | cTDCS | 54 | 594 | 92.4 | Female | Hemorrhagic | Fluent | Right | LSA, SFAT, WRC |
| 9 | cTDCS | 75 | 573 | 20.3 | Male | Ischemic | Fluent | Left | MIT, IS, ACT |
| 10 | cTDCS | 74 | 617 | 93.4 | Male | Ischemic | Fluent | Right | SFAT, WRC |
| 11 | cTDCS | 72 | 1004 | 70 | Male | Hemorrhagic | Nonfluent | Right | MRET, VNST, WRC |
| 12 | cTDCS | 68 | 1775 | 66.1 | Male | Both | Fluent | Right | MIT, VNST, SPT, IS, WRC |
| 13 | cTDCS | 41 | 869 | 69.1 | Male | Ischemic | Nonfluent | Right | SFAT, VNST, IS, WRC |
| 14 | cTDCS | 83 | 1679 | 31.5 | Male | Ischemic | Nonfluent | Right | IS, WRC |
| 15 | cTDCS | 70 | 2418 | 41 | Male | Ischemic | Nonfluent | Right | VNST, IS |
| 16 | cTDCS | 81 | 438 | 11.5 | Female | Ischemic | Nonfluent | Right | SFAT, IS, WRC, ACT |
| 17 | cTDCS | 53 | 822 | 13.7 | Male | Both | Nonfluent | Right | MIT, SFAT, IS, ACT |
| 18 | cTDCS | 77 | 398 | 76.5 | Female | Ischemic | Fluent | Right | VNST |
| 19 | cTDCS | 50 | 2362 | 14.7 | Male | Ischemic | Nonfluent | Right | MIT, IS, ACT |
| 20 | cTDCS | 61 | 365 | 16.9 | Male | Ischemic | Nonfluent | Right | MRET, IS, ACT |
| 21 | cTDCS | 71 | 1113 | 53.7 | Male | Ischemic | Fluent | Right | SFAT, WRC |
| 22 | cTDCS | 64 | 405 | 39.8 | Male | Ischemic | Nonfluent | Right | SFAT, IS, WRC, ACT |
| 23 | Sham | 59 | 957 | 94.00 | Male | Ischemic | Fluent | Right | LSA, MRET, SFAT |
| 24 | Sham | 69 | 446 | 91.70 | Male | Ischemic | Fluent | Right | SFAT, WRC |
| 25 | Sham | 81 | 5524 | 28.50 | Male | Ischemic | Nonfluent | Right | SFAT, IS |
| 26 | Sham | 46 | 1348 | 84.30 | Female | Both | Fluent | Right | LSA, SFAT, WRC |
| 27 | Sham | 69 | 6686 | 19.60 | Male | Hemorrhagic | Nonfluent | Right | LSA, IS |
| 28 | Sham | 32 | 596 | 98.60 | Male | Ischemic | Fluent | Right | LSA, SFAT, WRC |
| 29 | Sham | 59 | 3042 | 16.20 | Female | Both | Nonfluent | Right | SPT, WRC |
| 30 | Sham | 59 | 2440 | 71.90 | Male | Hemorrhagic | Fluent | Right | MRET, VNST, ACT |
| 31 | Sham | 60 | 1372 | 58.60 | Male | Ischemic | Fluent | Right | LSA, SPT, IS, WRC, ACT |
| 32 | Sham | 68 | 1871 | 78.80 | Male | Ischemic | Nonfluent | Right | MRET, SFAT, WRC |
| 33 | Sham | 66 | 2905 | 75.50 | Male | Ischemic | Fluent | Right | SFAT, IS, WRC |
| 34 | Sham | 45 | 1863 | 59.00 | Male | Ischemic | Nonfluent | Right | MIT, VNST, IS, WRC |
| 35 | Sham | 72 | 658 | 54.60 | Male | Hemorrhagic | Nonfluent | Right | MRET, SFAT, WRC |
| 36 | Sham | 56 | 4811 | 84.20 | Male | Ischemic | Fluent | Right | LSA, SFAT, WRC |
| 37 | Sham | 76 | 5927 | 63.50 | Female | Ischemic | Fluent | Right | SFAT, WRC |
| 38 | Sham | 70 | 6428 | 2.80 | Male | Ischemic | Nonfluent | Right | MIT, ACT |
| 39 | Sham | 59 | 2713 | 82.90 | Female | Ischemic | Fluent | Right | VNST, WRC |
| 40 | Sham | 62 | 5945 | 52.90 | Male | Ischemic | Nonfluent | Right | VNST, WRC, ACT |
| 41 | Sham | 78 | 6595 | 40.40 | Male | Ischemic | Nonfluent | Right | SFAT, WRC |
| 42 | Sham | 69 | 381 | 98.00 | Male | Ischemic | Fluent | Right | LSA, SFAT, WRC |
| 43 | Sham | 70 | 1804 | 65.10 | Male | Ischemic | Nonfluent | Right | SFAT, WRC |
| 44 | Sham | 63 | 412 | 74.40 | Male | Ischemic | Fluent | Left | MRET, WRC |
| 45 | Sham | 76 | 433 | 16.10 | Female | Ischemic | Nonfluent | Right | SFAT, IS |
| 46 | Sham | 67 | 1231 | 22.90 | Male | Ischemic | Nonfluent | Right | MIT, SFAT |
| 47 | Sham | 70 | 369 | 73.90 | Male | Ischemic | Fluent | Right | MRET, WRC |

Abbreviations: TSI: Time Since Injury; AQ: Aphasia Quotient; MIT: Melodic Intonation Therapy; LSA: Language Stimulation Approach; MRET: Modified Response Elaboration Training; SFAT: Semantic Feature Analysis Treatment; VNST: Verb Network Strengthening Treatment; SPT: Sound Production Treatment; IS: Integral Stimulation; and WRC: Word Retrieval Cuing Strategies
